# From the Cochrane Library: Interventions for Hidradenitis Suppurativa

**DOI:** 10.2196/29966

**Published:** 2022-03-11

**Authors:** Jalal Maghfour, Torunn Sivesind, Vincent Piguet, Robert Dellavalle, John R Ingram

**Affiliations:** 1 Photomedicine and Photobiology Research Department of Dermatology Henry Ford Health System Detroit, MI United States; 2 Dermatology Department University of Colorado Aurora, CO United States; 3 University of Toronto Toronto, ON Canada; 4 Dermatology Department Glamorgan House University Hospital of Wales Cardiff United Kingdom; 5 Division of Infection and Immunity Cardiff University Cardiff United Kingdom

**Keywords:** hidradenitis suppurativa, quality of life, outcome measures, heterogenetity in HS research, dermatology, comorbidities, treatment interventions, review

Hidradenitis suppurativa (HS) is a debilitating chronic inflammatory skin disorder with an estimated worldwide prevalence of 0.03% to 4% [1]. HS is strongly associated with metabolic and chronic inflammatory comorbidities [2], and there is increasing evidence demonstrating a link between HS and psychiatric comorbidities [2]. Psychiatric disorders are known to strongly affect patients’ quality of life [2]. Despite the various treatment interventions—from oral antibiotics to systemic agents such as biologics—therapeutic management of HS continues to be a challenge, highlighting the need to incorporate an evidence-based review of the interventions available. A 2015 Cochrane review [3] and its 2017 updated version [4] offered a comprehensive overview of the evidence regarding treatment interventions of HS and the impact on patients through the use of a validated instrument, Dermatology Life Quality Index (DLQI). In this synopsis, we provide a summary integrating evidence derived from the original review (2015), along with its updated and abridged 2017 version [3,4].

A total of 12 randomized controlled trials (RCTs; n=612; mean trial period 16 weeks) met the authors’ inclusion criteria, with the primary outcomes being DLQI and adverse events (AEs). Of 12 RCTs, 4 (33%) evaluated efficacy of anti–tumor necrosis factor (TNF) alpha (anti–TNF-α) agents, 1 (8.3%) assessed surgical intervention, and 3 (25%) discussed the efficacy of topical and oral medications; the remaining 4 (33%) studies explored utility of intense pulsed light (IPL), neodymium-doped yttrium aluminum garnet (Nd:YAG) laser, methylene blue topical gel photodynamic therapy, and staphage lysate. The quality of evidence was based on the *Grading of Recommendations, Assessment, Development and Evaluation* (*GRADE*) framework; the level of certainty for each included intervention is summarized in Table 1.

The level of certainty for infliximab (IFX), weekly adalimumab, and etanercept is moderate, while the level of certainty for biweekly adalimumab is high [3,4]. With regard to primary outcomes, all studies discussed, in varying degrees of detail, AEs—notably, AEs were difficult to assess in the included studies due to small numbers of participants and short study time frames. One study participant receiving biologic therapy with IFX experienced hypertension requiring hospitalization. Only 5 articles, which evaluated the efficacy of anti-TNF-α, provided DLQI results [3,4]. Among the remaining 8 studies [3], various scoring instruments (Participant/Physician Global Assessment, pain score, hidradenitis severity score, duration of remission) were used and were categorized by the authors as secondary outcomes.

**Table 1:** Quality of evidence for the included trials.

Trial intervention	Quality of evidence
Anti–TNF^a^-α (biweekly adalimumab, etanercept, infliximab) vs placebo	Moderate quality
Weekly adalimumab	High quality
Gentamicin sponge prior to closure vs primary closure alone	Moderate quality
Oral ethinylestradiol/oral norgestrel vs oral ethinylestradiol/cyproterone acetate	Moderate quality
IPL^b^ laser vs no treatment	Low quality
Nd:YAG^c^ laser vs topical control	Very low quality
Niosomal methylene blue gel PDT^d^ vs free methylene blue gel PDT	Low quality
Staphage lysate^e^ vs placebo broth	Moderate quality

^a^TNF: tumor necrosis factor.

^b^IPL: intense pulsed light.

^c^Nd:YAG: neodymium-doped yttrium aluminum garnet.

^d^PDT: photodynamic therapy.

^e^Although there was moderate evidence for the use of staphage lysate, this form of intervention is not routinely available.

Weekly adalimumab (ADA) 40 mg appeared effective for the treatment of moderate-severe HS [2,3]. Compared to placebo, ADA resulted in a statistically significant improvement of DLQI. Although each study evaluating weekly ADA resulted in a significant improvement in DLQI of at least 5 points, the difference in DLQI score between those treated with ADA group versus placebo was only 2.8 (95% CI 3.67-1.95) [3]. As such, the improvement may not be clinically relevant, given that the minimal clinically important difference (MCID) of the DLQI is an improvement of 4 points from baseline. However, it is important to note that DLQI is not specific to HS, and the use of newly developed and validated HS-specific quality of life (QoL) instruments (eg, HiSQOL) may be better suited to capture changes in QoL among patients with HS.

Similar to weekly ADA, a single RCT evaluating the efficacy of 5 mg/kg IFX demonstrated a significant improvement in DLQI (8.4 points) compared to placebo (*P*=.03). Although these results are promising, they should be interpreted with caution given that the quality of evidence supporting the use of IFX for improving patients’ quality of life is “moderate”—meaning that future studies will likely have an impact on the estimated effect. Biweekly ADA and etanercept 50 mg failed to improve DLQI among treated patients. Anakinra, an interleukin 1 (IL-1) antagonist, resulted in a significant reduction in disease activity score (*P*=.04). However, there was no significant improvement in DLQI (*P*=.08).

With the addition of its 2017 update, this Cochrane review [3,4] demonstrated the high-quality evidence that exists for the use of weekly ADA for the treatment of moderate to severe HS. Recently published data from the PIONEER studies provide further support for the safety and efficacy of weekly ADA [5,6]. Although DLQI was the primary end point in this study, there are limited studies that have explored its validity in HS [7]. As such, there is a need to adopt a validated core outcome set for HS when testing the safety and efficacy of new therapies in RCTs. Nevertheless, this review highlights the limited evidence, primarily due to underpowered studies, that exists for the use of other treatment modalities in patients with HS; thus, additional well-designed RCTs are warranted.
